# 

**Published:** 1951-07

**Authors:** 


					Published under the auspices of the Bristol medico-chirurgical society
THE BRISTOL
MEDICO - CHIRURGICAL
JOURNAL
A Journal of the Medical Sciences for the
WEST OF ENGLAND
AND SOUTH WALES
Editor:
E. WATSON-WILLIAMS, M.C., M.D., Ch.M., F.R.C.S.
with whom are associated
G. E. F. SUTTON, M.i
J. H. MIDDLEMISS,
! N. S. CRAIG,
Locil Correspondet\
Bath?A. D. BATEMAN,
Exeter?L.
Gloucester?R. F.
Newport?J. L. D. WILLIAMS, M.B., F.R.C.S.
Plymouth?C. R. CROFT, D.M., M.R.C.P. Taunton?Dr. M. McALLEY, D. M.R.
Torquay?Dr. A. ROBINSON THOMAS, D.M.R.E.
Truro?Dr. F. D. M. HOCKING, F.R.I.C. Yeovil?J. A. VERE NICOLL, F.R.C.S.
BRISTOL t
J. W. ARROWSMITH LTD., QUAY. STREET AND SMALL STREET
JULY * 1951
V Lxvm. No. 247. Price i FOUR SHILLINGS net.

				

